# Benznidazole/Itraconazole Combination Treatment Enhances Anti-*Trypanosoma cruzi* Activity in Experimental Chagas Disease

**DOI:** 10.1371/journal.pone.0128707

**Published:** 2015-06-15

**Authors:** Tassiane Assíria Fontes Martins, Lívia de Figueiredo Diniz, Ana Lia Mazzeti, Álvaro Fernando da Silva do Nascimento, Sérgio Caldas, Ivo Santana Caldas, Isabel Mayer de Andrade, Isabela Ribeiro, Maria Terezinha Bahia

**Affiliations:** 1 Laboratório de Doenças Parasitárias, Escola de Medicina & Núcleo de Pesquisas em Ciências Biológicas, Universidade Federal de Ouro Preto, Campus Universitário, Morro do Cruzeiro, Ouro Preto, Minas Gerais, Brazil; 2 Fundação Ezequiel Dias, Belo Horizonte, Minas Gerais, Brazil; 3 Departamento de Patologia e Parasitologia, Instituto de Ciências Biomédicas, Universidade Federal de Alfenas, Alfenas, Minas Gerais, Brazil; 4 Drugs for Neglected Disease *initiative* (DND*i*), 1202 Geneva, Switzerland; State University of Campinas, BRAZIL

## Abstract

The nitroheterocyclic drugs nifurtimox and benznidazole are first-line drugs available to treat Chagas disease; however, they have limitations, including long treatment courses and toxicity. Strategies to overcome these limitations include the identification of new drugs with specific target profiles, re-dosing regimens for the current drugs, drug repositioning and combination therapy. In this work, we evaluated combination therapy as an approach for optimization of the current therapeutic regimen for Chagas disease. The curative action of benznidazole/itraconazole combinations was explored in an established infection of the mice model with the *T*. *cruzi* Y strain. The activities of the benznidazole/itraconazole combinations were compared with the results from those receiving the same dosage of each individual drug. The administration of benznidazole/itraconazole in combination eliminated parasites from the blood more efficiently than each drug alone. Here, there was a significant reduction of the number of treatment days (number of doses) necessary to induce parasitemia suppression with the benznidazole/itraconazole combination, as compared to each compound administered alone. These results clearly indicate the enhanced effects of these drugs in combination, particularly at the dose of 75 mg/kg, as the effects observed with the drug combinations were four times more effective than those of each drug used alone. Moreover, benznidazole/itraconazole treatment was shown to prevent or decrease the typical lesions associated with chronic experimental Chagas disease, as illustrated by similar levels of inflammatory cells and fibrosis in the cardiac muscle tissue of healthy and treated mice. These results emphasize the importance of exploring the potential of combination treatments with currently available compounds to specifically treat Chagas disease.

## Introduction

American trypanosomiasis, also known as Chagas disease, is a protozoan infection caused by *T*. *cruzi*. This human infection is endemic in countries of Central and South America. Despite vector control programs and other measures taken in blood banks and maternity hospitals, approximately eight million people are estimated to be infected worldwide and over 25 million are at risk [1].

For most of the past 40 years, the array of therapeutics options for the treatment of Chagas disease has been extremely limited. Current specific chemotherapy, which is based on the nitroheterocyclic compounds nifurtimox and benznidazole, has limitations due to side effects, long treatment regimens and some degree of variation in the sensitivity among parasite populations[2,3,4]. Strategies to overcome these limitations include the identification of new drugs with specific target profiles, re-dosing regimens for the current drugs, drug repositioning and combination therapy [5]. Among these strategies, drug repositioning and re-dosing regimens for the current drugs, as monotherapy or in combination therapy, are the quickest interventions to improve Chagas disease therapy.

Drug repositioning has been successful at identifying C14-α-demethylase (CYP51) inhibitors as anti-*T*. *cruzi* agents [6]. A number of these inhibitors have been reported to exhibit potent anti-*T*. *cruzi* activity in experimental animals. A phase II clinical trial to investigate the efficacy and safety of posaconazole and E1224 were recently completed, and reports indicate that both compounds had little to no sustained efficacy in treating patients in the chronic phase of Chagas disease as a single medicine [7,8]. These results highlight the need to investigate alternative dosing regimens and possible combination therapies to improve the efficacy of Chagas disease treatment.

Combination therapies for the treatment of Chagas disease have increasingly been advocated as a way of enhancing treatment efficacy and tolerance. Consensus has grown in favor of the use of combination regimens for infectious diseases over the past few years for several reasons. Combining drugs from different chemical classes could reduce drug doses and/or treatment duration, resulting in fewer side effects. This strategy could also reduce the overall costs, providing a more cost-effective option. Finally, combination therapy could improve treatment efficacy for life-threatening acute *T*. *cruzi* infections in humans, such as those of oral, congenital or reactivated Chagas disease patients.

Studies investigating interactions among sterol biosynthesis inhibitors that act at different steps of its biosynthesis pathway have shown synergistic effects against *T*. *cruzi* [9,10]. Other studies specifically focused on interactions between ketoconazole and benznidazole or posaconazole and benznidazole have shown enhancement in the efficacy of chemotherapy for an experimental infection when these drugs are used in combination [11,12]. More recently, Moreira da Silva [13] showed that the administration of benznidazole in combination with itraconazole in mice induces lower elimination of benznidazole (prolonged half-life) using the HPLC-UV method and determined an accumulation profile in this animal model. The authors suggest that this effect may contribute to improving the therapeutic efficacy of these compounds when administered in combination against *T*. *cruzi* infection. Itraconazole has been used in humans as an efficient antimycotic without severe side effects [14]. A number of studies have been shown the *in vivo* curative activity of itraconazole in human [15] and in experimental animals [16]. Others have shown a suppressive, but not a curative activity, of itraconazole [17].

Considering these antecedents, this study was designed to investigate the efficacy of benznidazole in combination with itraconazole against *T*. *cruzi* in an experimental murine model of acute Chagas disease to support the potential clinical evaluation of such combination therapies.

## Materials and Methods

### Ethics statements

All procedures and experimental protocols were conducted in accordance with the guidelines issued by the Brazilian College of Animal Experimentation (COBEA) and approved by the Ethics Committee in Animal Research at Universidade Federal de Ouro Preto (number 2009/16).

### Parasite

The *T*. *cruzi* Y strain (DTU II), which was previously characterized by Filardi & Brener [18] as partially resistant to benznidazole, was used in the present study. The original isolate was preserved in its trypomastigote form in liquid nitrogen, periodically transferred to mice and then refrozen with full retention of its biological and drug susceptibility characteristics.

### Drugs

The following drugs were commercially purchased from or provided by their respective pharmaceutical companies:

- Benznidazole- 2-nitro-imidazole-(N-benzil-2-nitro-1-imidazoleacetamide (produced by LAFEPE, Brazil).
- Itraconazole- 4-(4-(4-(4-((2-(2,4-dichlorophenyl)-2-(1H-1,2,4-triazol-1-ylmethyl)-1,3-dioxolan-4-yl)methoxy)phenyl)-1-piperazinyl)phenyl)-2,4-dihydro-2-(1-methylpropyl) (Sporanox, Janssen-Cilag, produced by Brainfarma).
- Cyclophosphamide (N,N-bis(2-chloroethyl)-1,3,2-oxazaphosphinan-2-amine 2-oxide; Genuxal, Asta Medica Oncologica).

### *In vivo* assays

Female Swiss mice (18–22g) were obtained from the Animal Facility at the Federal University of Ouro Preto, Minas Gerais, Brazil and maintained in a temperature-controlled room with access to water and food *ad libitum*. The animals were inoculated intraperitoneally with 5,000 blood trypomastigotes *of T*. *cruzi* Y strain. After four days, tail blood was examined for the presence of parasites. The mice were subjected to a specific treatment when *T*. *cruzi* was detected microscopically.

### Dose-response experiment

Infected animals were divided into groups of 10 and received drugs (benznidazole and itraconazole) at doses of 50, 75 and 100 mg/kg of bodyweight (mg/kg). The compounds were suspended in distilled water using 4% methyl-cellulose (Sigma), and each animal received 0.2 mL of drug suspension daily by gavage for 20 consecutive days. The optimal dose (dose effective in curing mice infected with different *T*. *cruzi* strains) of benznidazole and itraconazole for mice infection treatment was 100 mg/kg [18,16].

### Drug combination experiment

The drug combinations consisted of benznidazole plus itraconazole at the following dosages: 50, 75 or 100 mg/kg of benznidazole in combination with the same doses of itraconazole. Drugs were administered for 20 consecutive days upon detection of parasitemia, which occurs at the 4^th^ day post-inoculation.

### Assessment of parasitological cure

Parasitological cure was evaluated following the methodology standardized by Caldas *et al*, [19] based on a battery of three independent tests: fresh blood examination before and after cyclophosphamide immunosuppression (CyI), followed by PCR assays performed on blood samples from mice with negative parasitemia at the 1^st^ and 6^th^ month after treatment and anti-*T*. *cruzi* IgG levels evaluated at the 1^st^ month after treatment. Animals showing negative results in all tests were considered cured.

### Fresh blood examination

Parasitemia was evaluated during and up to the 30th day post-treatment to detect the natural reactivation of infection. Animals that did not exhibit evidence of reactivation after treatment were treated with CyI, which consists of three cycles of 50 mg of cyclophosphamide/kg of body weight for four consecutive days, with intervals of three days between each cycle. Parasitemia in these animals was evaluated during the CyI cycles and for 10 days after immunosuppression.

### PCR assay

Mice were bled from the orbital venous sinus, and 200 μL of blood was collected at the 1^st^ and 6^th^ month after treatment. PCR was performed only in samples from negative animals as determined by fresh blood examination. DNA extraction and PCR were performed according to Gomes *et al*. [20] with some modifications. The primers used for the parasite minicircle amplification were the following: S35 5’-AAATAATGTACGGG(T/G)GAGATGCATGA-3’ and S36 5’-GGGTTCGATTGGGGTTGGTGT-3’ [21]. Thirty-five amplification cycles were carried out in a Research Programmable Thermal Controller (MJ Research, model PTC-150). The cycles consisted of an initial denaturation of 5 minutes at 95°C, followed by 35 cycles of 1 minute each at 95°C for denaturation, 1 min at 65°C for primer annealing and 1 minute at 72°C for primer extension. Five microliters of the PCR product was analyzed by electrophoresis on a 6% polyacrylamide gel and visualized by silver staining. Positive and negative blood samples and reagent controls were processed in parallel in each assay, and all experiments were conducted under controlled conditions. To avoid contamination, DNA extraction, mixing, and electrophoresis were performed in separate, delineated areas. To confirm the absence of inhibition factors, an internal control corresponding to a segment of the murine TNF-α gene was amplified [22].

### Influence of the specific treatment on IgG levels

Blood from treated mice was collected from the orbital venous sinus (500 μl) at the 1^st^ month after treatment. *T*. *cruzi*-specific antibodies were detected by the technique described by Voller *et al* [23]. Enzyme-linked immunosorbent assay plates were coated with *T*. *cruzi* antigen prepared from alkaline extraction from the Y strain during exponential growth in LIT medium. Peroxidase-conjugated anti-mouse IgG antibody (Sigma Chemical Co.) was used. The mean absorbance values for the 10 negative control samples were used to determine the reactivity index value by dividing the absorption value (O.D. value) of each serum sample by the mean value of the differential controls sample.

### Myocardial Tissue Assessment

For morphometric analysis, mice were sacrificed six months after treatment. Animals were sacrificed by cervical dislocation, and heart tissues were fixed with 10% formalin and embedded in paraffin. Blocks were cut into 4 μm-thick sections and stained with either hematoxylin-eosin (H&E) to detect inflammation or Masson's trichrome stain to assess fibrosis. Twenty fields from each H&E- or Masson's trichrome-stained section were chosen at random and evaluated at 40x magnification, resulting in the analysis of a total area of 74931 μm^2^ of myocardium. Images were obtained using a Leica DM 5000 B microchamber (version 2.4.0 R1; Leica Application Suite, United Kingdom) and processed using Leica Qwin (version 3) image analyzer software. The fibrotic areas were quantified using the image segmentation function. All pixels with blue hues in the Masson's trichrome-stained sections were selected to build a binary image, and the total area occupied by connective tissue in uninfected and *T*. *cruzi*-infected mice was subsequently calculated.

### Statistical analysis

To evaluate the dose-dependent effect in reducing the parasitemia, the data were converted using a logarithmic transformation and tested using Pearson’s correlation coefficient. The variations in the levels of parasitemia among the animals treated with each drug alone or in combination were evaluated using analysis of variance, and comparison among groups was performed using Tukey's Multiple Comparison Test. The results of the morphometric analysis were expressed as the average ± standard deviation and analyzed using the Mann-Whitney test. Differences were considered significant when p < 0.05.

## Results

**Fig 1:**
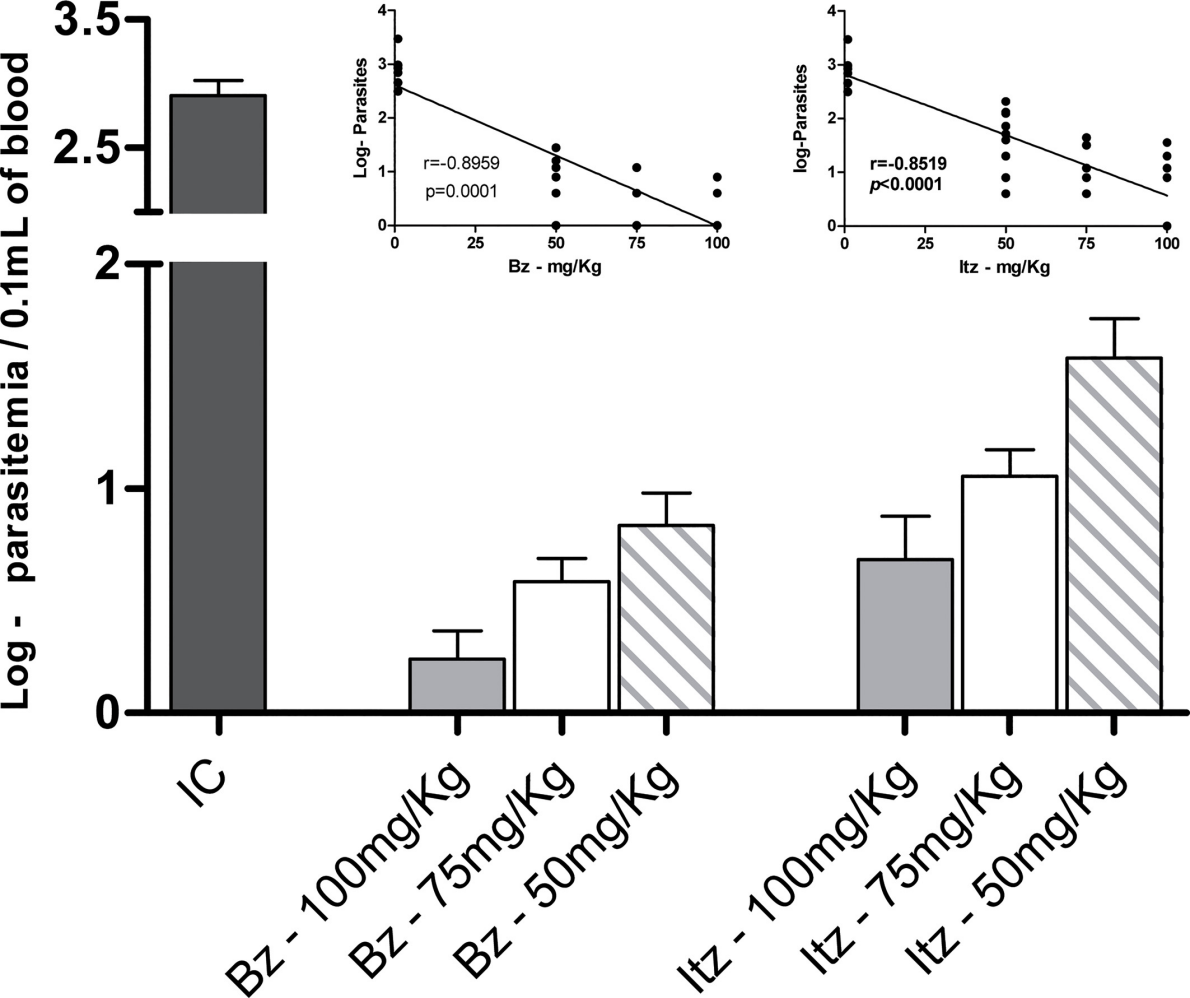
Effects of benznidazole and itraconazole treatment on parasitemia levels. Maximum number of trypomastigotes detected in the peripheral blood of mice infected with *Trypanosoma cruzi* Y strain and treated with daily doses 50, 75 or 100 mg/kg bodyweight (mg/kg) of benznidazole (Bz) or itraconazole (Itz) for 20 consecutive days and those in the infected control group (IC). Insert indicates Pearson correlation analysis between drug dose and parasitemia levels.

The parasitemia levels and mortality rates of animals infected with the *T*. *cruzi* Y strain were assessed. All untreated animals had higher levels of parasitemia that peaked on the 8^th^ day post-infection, and mortality occurred on average at 15 days post-infection (data not shown). The effects of suboptimal doses (50 and 75 mg/kg) of each drug alone on the evolution of the infection in mice were then evaluated and compared with those observed in mice treated with an optimal dose (100 mg/kg) of benznidazole and itraconazole. The optimal dose of each compound lessened mortality and proliferation of *T*. *cruzi* in all treated mice (Table 1). For the suboptimal doses of itraconazole, suppression of parasitemia was verified only in those animals treated with 75 mg/kg. Parasitemia suppression was observed in animals treated with benznidazole, regardless of dosage. Given these results, the influence of the therapeutic scheme on the course of the acute infection of mice was evaluated considering the parasitemia levels and survival rate of animals after specific treatment. The analysis of parasitemia post-treatment showed that all compounds had a dose-dependent effect. The optimal dose of each compound was able to markedly reduce parasitemia (Fig 1), as demonstrated by the significantly smaller (p<0.05) log of maximum parasitemia based on which animals treated with benznidazole or itraconazole were compared with those receiving suboptimal doses (Fig 1). Similarly, the survival of itraconazole-treated animals also exhibited a dose-dependent curve, and only mice receiving the optimal dose stayed alive (100% survival) until 30 days post-treatment. In contrast, those animals receiving suboptimal doses of benznidazole exhibited 100% survival (Table 1).

**Table 1:** Efficacy of benznidazole (Bz) and itraconazole (Itz) treatments for 20 days (monotherapy or combination) in murine model of acute *Trypanosoma cruzi* infection.

Experimental groups^1^	Parasitemia clearance (doses)^2^	Negative Results in FBE or PCR assays^3^ (%)	Number of surviving/ total number of animals
Unifected	-	10/10 (100%)	10/10 (100%)
Untreated	0/10	0/10 (0%)	0/10 (0%)
Bz 100 mg/kg/day	10/10 (1.5±0.32)	7/10(70%)	10/10 (100%)
Itz 100 mg/kg/day	10/10 (2.9±3.76)	0/10 (0%)	10/10 (100%)
**Bz + Itz (100 + 100 mg/kg/day)**	**10/10 (1.0+0)**	**8/10 (80%)**	**10/10 (100%)**
Bz 75 mg/kg/day	10/10 (1.5±0.70)	2/10 (20%)	10/10 (100%)
Itz 75 mg/kg/day	3/10 (13±4.58)	0/10 (0%)	4/10 (40%)
**Bz + Itz (75 + 75 mg/kg/day)**	**10/10 (1.0+0)**	**8/10 (80%)**	**10/10 (100%)**
Bz 50 mg/kg/day	10/10 (4.2±3.73)	0/10 (0%)	10/10 (100%)
Itz 50 mg/kg/day	0/10	0/10 (0%)	4/10 (40%)
**Bz + Itz (50 + 50 mg/kg/day)**	**10/10 (1.2+0.42)**	**2/10 (20%)**	**9/10 (90%)**

^1^ Swiss female weight 18 to 22g were inoculated with 5x103 trypomastigotes (Y strain), treatment was initiated at 4 days after inoculation followed by 20 days and it was administered per oral route.

^2^ Mean of doses (or days of treatment) required to induce the parasitemia supression.

^3^ Fresh blood examination (FBE) before and after cyclophosphamide immunosuppression and PCR assays performed at 1st and 6th month post-treatment.

Then, the activities of the benznidazole/ itraconazole combinations were compared with the results from those receiving the same dosage of each individual drug. The benznidazole/itraconazole combination administered eliminated parasites from the blood more efficiently than each drug alone. Here, the beneficial effect of drug combinations was demonstrated by the reduction of the number of treatment days (number of doses) necessary to induce parasitemia suppression in relation to each compound administered alone (Table 1). These results clearly indicate the enhanced effects of the combined action of the drugs, particularly at the dose of 75 mg/kg, as the effects observed with the drug combinations were four times more effective than those of each drug used alone. In other words, itraconazole administered at 75mg/kg cured none of the Y strain-infected mice and benznidazole at 75mg/kg induced just 20% of cure, while benznidazole/itraconazole treatment at the same doses cured 80% animals.

**Fig 2:**
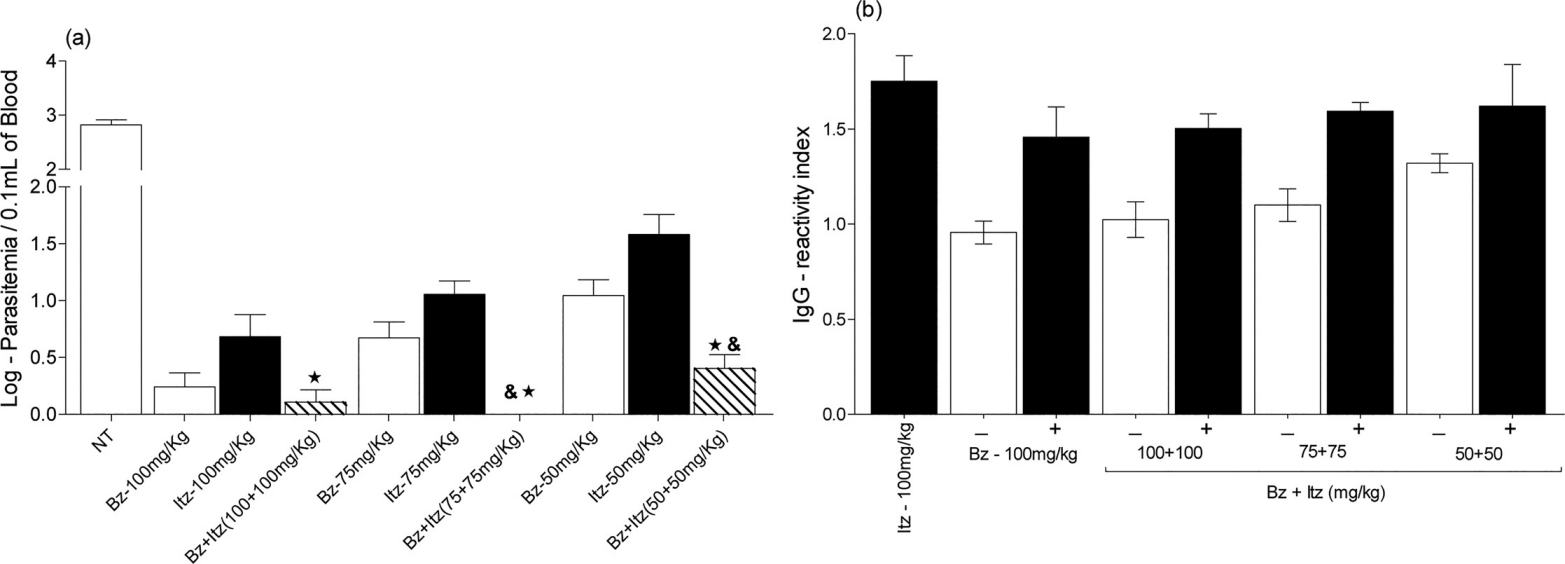
Anti-*Trypanosoma cruzi* activity of benznidazole/itraconazole combination therapy. Mice were inoculated with 5,000 *T*. *cruzi* Y strain trypomastigotes by the intraperitoneal route and treated with daily doses 50, 75 and 100 mg/kg of bodyweight (mg/kg) of benznidazole (Bz) or itraconazole (Itz) alone or in combination for 20 consecutive days. (a) Maximum number of trypomastigotes detected in the peripheral blood of treated mice up to 30 days post-treatment. (b) IgG antibodies in treated mice by ELISA up to 30 days post-treatment. The results are shown as the reactivity index value, which was obtained by dividing the absorption value (O.D. value) of each serum sample by the mean value of the differential control uninfected sample. (-) negative results in fresh blood examination and PCR assay, (+) positive results in fresh blood examination and/or PCR assay, and **&** significant difference relative to benznidazole treatment administered alone at the same dose; ★ significant relative to itraconazole treatment administered alone at the same dose.

Additionally, the analysis of the log of the maximum parasitemia detected in the blood of each treated and uncured animal showed significant efficacy of the combined treatment in reducing parasite load relative to the efficacy of each drug administered alone (Fig 2A). The inhibitory effects observed were statistically different at all drug concentrations.

Comparative analysis of specific *T*. *cruzi* antibodies detected in blood samples collected at the 1^st^ month post-treatment was performed. For these analyses, experimental animals were classified based on the results of the parasitological and PCR evaluations: (-) treated mice that had negative parasitological and PCR results, and (+) treated mice that had positive parasitological and PCR results. A concordance among parasitological, molecular assays and serological tests was observed. All animals with negative results in the fresh blood examination and PCR had reactivity index values near 1; in other words, all cured animals had IgG antibodies levels similar to those of healthy mice (Fig 2B).

**Fig 3:**
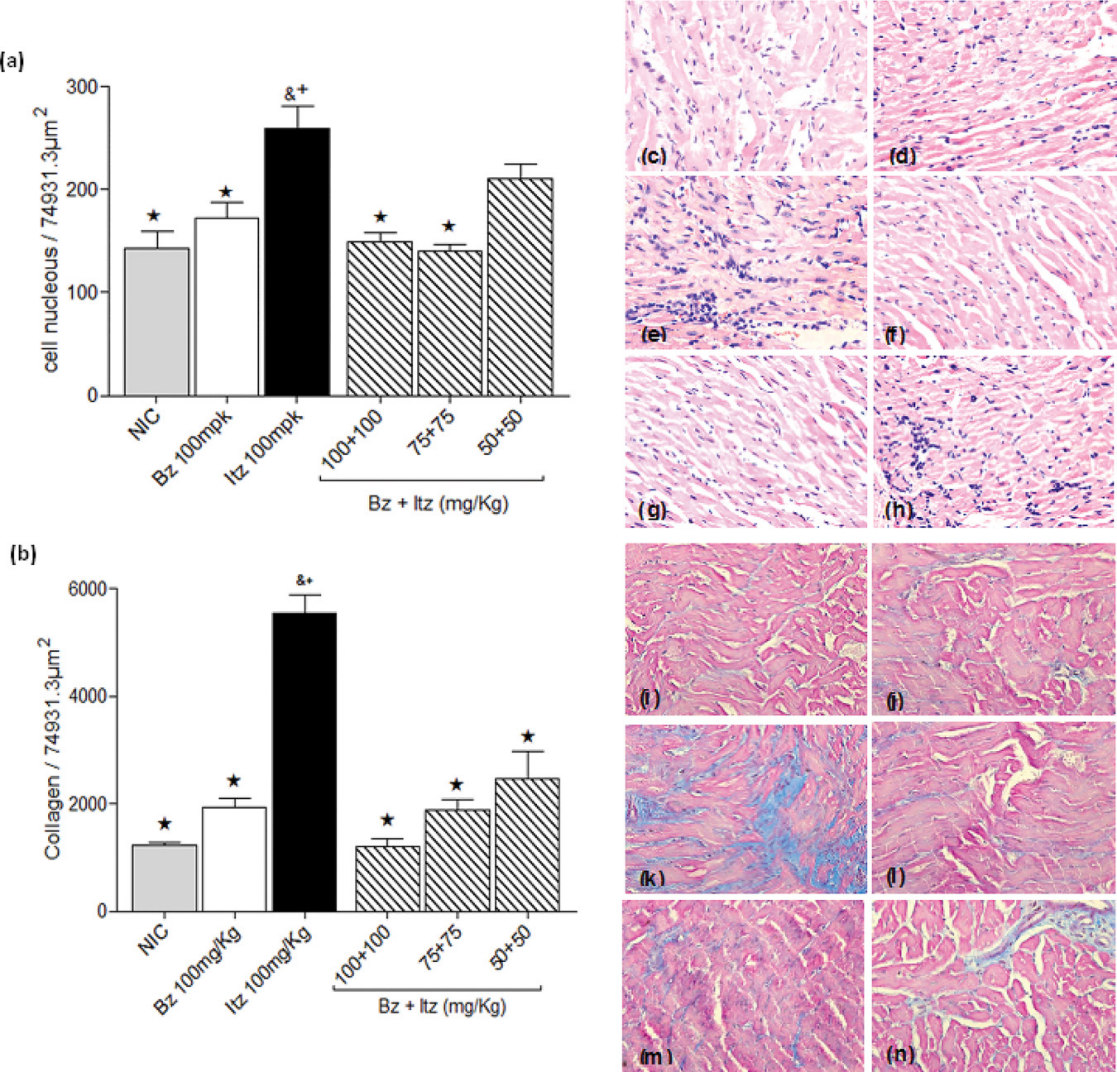
Effect of benznidazole/itraconazole combination treatment on chronic cardiac lesions. Mice were inoculated with 5,000 *Trypanosoma cruzi* Y strain trypomastigotes by the intraperitoneal route and were treated with daily doses 100 mg/kg of bodyweight of benznidazole (Bz) or itraconazole (Itz) alone or in combination at doses of 50, 75 and 100 mg/kg of bodyweight of each drug for 20 consecutive days. Animals were euthanized six months post treatment. Uninfected control (NIC) groups were also evaluated. (a) Myocardial inflammatory cell count in heart muscle of mice, and (b) Fibrotic areas in the heart muscle of treated and uninfected mice. (c-n) Analysis of histological sections of hearts from mice, 40 x magnification: (c-h) Hematoxylin-Eosin staining for inflammation assessment and (i-n) Masson’s trichrome stain for fibrosis assessment. (c and i) Myocardial sections from healthy mice; (d and j) from infected benznidazole 100 mg/kg-treated; (e and k) from infected itraconazole 100 mg/kg-treated; (f and l) from benznidazole (100 mg/kg)/itraconazole (100 mg/kg)-treated; (g and m) from benznidazole (75 mg/kg)/itraconazole (75 mg/kg)-treated; (h and n) benznidazole (50 mg/kg)/itraconazole (50 mg/kg)-treated. & significant difference in relation to uninfected control. ★+Significant difference in relation to itraconazole (★) and benznidazole (+) treatments administered at the optimal dose (100 mg/kg).

To evaluate the efficacy of early treatment in preventing the development of chronic lesions in mice infected with the Y strain, a quantitative analysis of the inflammation and fibrotic area in the cardiac muscle tissue of treated and non-infected mice was performed at 6 months of treatment. The effects of combined treatment (50, 75 and 100 mg/kg) were evaluated and compared with those observed in mice treated with the optimal dose (100 mg/kg) of benznidazole or itraconazole and those observed in healthy animals. The intensity of inflammatory infiltration and the fibrotic area in the hearts of mice infected with the Y strain and treated with an optimal dose of benznidazole was very similar to that of healthy animals. In contrast, the optimal dose of itraconazole did not reduce the intensity of heart inflammation or the fibrotic area in infected mice (Fig 3A and 3B). Benznidazole/itraconazole combination treatment was able to prevent or lessen the typical lesions in heart tissue associated with the chronic phase of experimental *T*. *cruzi* infection, as illustrated by similar levels of inflammatory cells and fibrotic areas observed in the heart muscle tissue of treated and healthy mice. Moreover, mice treated with benznidazole/itraconazole had fewer inflammatory cells and a smaller fibrotic area than those of itraconazole-treated mice (Fig 3).

## Discussion

Various studies have shown that new therapies, including combinations of drugs with different mechanisms of action, may improve the efficacy of treatments and/or reduce the adverse effects [11,12]. Combination therapy is used to treat a number of infectious diseases, such as toxoplasmosis, malaria, tuberculosis and AIDS [24,25,26,27]. Previous studies have shown that combinations of nitroheterocycles and CYP51 inhibitors compounds, such as benznidazole and posaconazole [12], at suboptimal doses or shorter treatment durations have equivalent or superior efficacy compared to the individual drugs given at their optimal doses and full treatment length in a murine model of Chagas disease.

Recently, Moreira da Silva et al [13] showed that itraconazole can alter the pharmacokinetics of benznidazole after oral coadministration in mice, increasing the volume of distribution and elimination half-life of benznidazole. The authors suggest that this alteration of the pharmacokinetic profile of benznidazole could be therapeutically useful. Considering this hypothesis, we investigated the trypanocidal activity of itraconazole combined with benznidazole during acute *T*. *cruzi* infection in mice. To better evaluate the efficacy of the combined therapy, the effects of the monotherapies at the same doses were also determined. Our results confirm findings from previous reports showing the high levels of *in vivo* activity of itraconazole and benznidazole, which confer almost complete protection against death to infected mice when used at optimal doses (100 mg/kg) [13,16]. Treatments of infected animals with doses lower than those indicated above were unable to induce parasitological cures (benznidazole) or provide parasitemia suppression (itraconazole), confirming the dose-dependent anti-*T*. *cruzi* activity of these drugs. Additionally, the beneficial effect of the combined treatment was evidenced by increased effectiveness in suppressing and/or reducing the parasitism of the drugs administered at suboptimal dosages compared to the effects of optimal doses of each compound administered alone. The trypanocidal activity of benznidazole/itraconazole was confirmed by further reductions in the parasite load observed in treated and uncured animals in relation to those observed in animals that received each drug alone. The reasons for the different cure rate detected in benznidazole/itraconazole therapy could be interpreted in terms of the different mechanisms of action, efficacy and time-to-kill of the two drugs. It suggests that benznidazole would lead to a rapid and significant reduction of parasite biomass for subsequent action of itraconazole. The unique pharmacokinetic properties of CYP51 inhibitors in mammal tissue, such as a long terminal half-life and large volume of distribution may contribute to the control of infection through its inhibitory effect on parasite replication. Therefore, the ability of itraconazole to alter the pharmacokinetic profile of benznidazole could be responsible, at least partially, for the enhanced efficacy of this drug combination [13].

Furthermore, our data show that the combination benznidazole/itraconazole (75 mg/kg) treatment was able to induce similar levels of cure (80%) compared with the effect of benznidazole monotherapy at the optimal dose (70%); this combination represents the possibility of 25% reduction of the doses of each of the compounds used as monotherapy. In addition, suboptimal doses of benznidazole/itraconazole were effective in preventing cardiac lesions, which are characteristic of the chronic phase of infection, more efficiently than the optimal treatment with itraconazole and benznidazole in monotherapy, even when a parasitological cure was not observed. These results are consistent with the hypothesis that there is a positive correlation between the parasitic load and the intensity of inflammation and disease severity in humans and experimental animals [28,29,30,31]. Others studies have been demonstrated that a reduction in parasite load achieved by the effect of trypanocidal therapy has always a favorable effect on *T*. *cruzi* infection outcome [32,33,34]. These findings confirm previous studies in which lower levels of antibodies specific to *T*. *cruzi* antigens, as well a reduced tissue damage, were detected [34]. Taken together, the results of this initial study support the notion that the use of itraconazole in combination with benznidazole could reduce the doses needed to obtain the same trypanocidal effect and, consequently, should diminish the treatment’s side effects and cost. Considering these results, this therapeutic scheme could be evaluated as an alternative treatment option for the management of life-threatening acute *T*. *cruzi* infections in humans, such as those in congenital or reactivated Chagas disease patients.

The results of the present study demonstrate that suboptimal doses of benznidazole/itraconazole in combination therapy have equivalent or superior efficacies than these compounds given at their optimal doses in a murine model of acute Chagas disease, indicating a positive interaction with the concomitant treatment. Our results reinforce the importance of evaluating already-marketed compounds with synergistic effects. This approach may offer alternative treatment options with a more rapid turnaround time for clinical evaluation.

## References

[1] WHO. World Health Organization (2010) Media centre. In: Chagas disease (American trypanosomiasis). Fact sheet N°340.

[2] Guedes PM, Fietto JLR, Lana M, Bahia MT (2006) Advances in Chagas Disease Chemotherapy. Anti-Infective Agents in Medicinal Chemistry 5: 175–86.

[3] Soeiro MNC, De Castro SL (2009) *Trypanosoma cruzi* targets for new chemotherapeutic approaches. Expert Opin Ther Targets 13: 105–21. 10.1517/14728220802623881 19063710

[4] Urbina JA (2009) Ergosterol biosynthesis and drug development for Chagas disease. Mem Inst Oswaldo Cruz 1:311–18. 19753490 10.1590/s0074-02762009000900041

[5] Bahia MT, Diniz L de F, Mosqueira VC (2014) Therapeutical approaches under investigation for treatment of Chagas disease. Expert Opin Investig Drugs 23:1225–37. 10.1517/13543784.2014.922952 24855989

[6] Urbina JA (2010) Specific chemotherapy of Chagas disease: relevance, current limitations and new approaches. Acta Trop 115(1–2):55–68. 10.1016/j.actatropica.2010.04.004 19900395

[7] Molina I, i Prat JG, Salvador F, Treviño B, Sulleiro E, Serre N, et al (2014) Posaconazole versus benznidazole for chronic Chagas' disease. N Engl J Med 371:966. 10.1056/NEJMc1407914 25184871

[8] Torrico F. 2013. Annual Meeting of the American Society of Tropical Medicine and Hygiene. 10.4269/ajtmh.17-medals PMC5805085 29187271

[9] Urbina JA, Lazardi K, Larralde G, Aguirre T, Piras MM, Piras R (1988) Synergistic effects of ketoconazole and SF-86327 on the proliferation of epimastigotes and amastigotes of *Trypanosoma (Schizotrypanum) cruzi* . Ann N Y Acad Sci 544: 357–8. 3063176 10.1111/j.1749-6632.1988.tb40421.x

[10] Maldonado RA, Molina JT, Payares G, Urbina JA (1993) Experimental chemotherapy with combinations of ergosterol biosynthesis inhibitors in murine models of Chagas’ disease. Antimicrob Agents Chemother 37: 1353–9. 8328786 10.1128/aac.37.6.1353 PMC187965

[11] Araújo MSS, Martins-Filho OA, Pereira MÊS, Brener Z (2000) A combination of benznidazole and ketoconazole enhances efficacy of chemotherapy of experimental Chagas disease. J Antimicrob Chemother 45: 819–24. 10837436 10.1093/jac/45.6.819

[12] Diniz LF, Urbina JA, de Andrade IM, Mazzeti AL, Martins TA, Caldas IS, et al (2013) Benznidazole and Posaconazole in Experimental Chagas Disease: Positive Interaction in Concomitant and Sequential Treatments. PLoS Negl Trop Dis 7(8): e2367 10.1371/journal.pntd.0002367 23967360 PMC3744424

[13] Moreira da Silva R, Oliveira LT, Silva Barcellos NM, de Souza J, Lana M (2012) Preclinical monitoring of drug association in experimental chemotherapy of Chagas' disease by a new HPLC-UV method. Antimicrob Agents Chemother 56: 3344–8. 10.1128/AAC.05785-11 22450981 PMC3370797

[14] Van Cauteren H, Coussement W, Vandenbughe J, Klerin V, Vanparys PH, Marsboom R (1987) The toxicological properties of itraconazole Fromtling RA, ed. Recent Trends in the Discovery, Development and Evaluation of Antifungical Agents. Beerse, Belgium: Prous Science Publishing Co 101–108.

[15] Apt W, Aguilera X, Arribada A, Pérez C, Miranda C, Sánchez G, et al (1998) Treatment of chronic Chagas’ disease with itraconazole and allopurinol. Am J Trop Med Hyg 59:133–138. 9684641 10.4269/ajtmh.1998.59.133

[16] Toledo MJ, Bahia MT, Carneiro CM, Martins-Filho AO, Tibayrenc M, Barnabé C, et al (2003) Chemotherapy with benznidazole and itraconazole for mice infected with different *Trypanosoma cruzi* clonal genotypes. Antimicrob Agents Chemother 47: 223–30. 12499195 10.1128/AAC.47.1.223-230.2003 PMC149031

[17] Moreira AA, de Souza HB, Amato Neto V, Matsubara L, Pinto PL, Tolezano JE, et al (1992) Evaluation of the therapeutic activity of itraconazole in chronic infection, experimental and human, by *Trypanosoma cruzi* . Rev Inst Med Trop São Paulo 34:177–80 1340033

[18] Filardi LS, Brener Z (1987) Susceptibility and natural resistance of *Trypanosoma cruzi* strains to drugs used clinically in Chagas disease. Trans R Soc Trop Med Hyg 81:755–9. 3130683 10.1016/0035-9203(87)90020-4

[19] Caldas S, Santos FM, de Lana M, Diniz LF, Machado-Coelho GLL, Veloso VM, et al (2008) *Trypanosoma cruzi*: acute and long term infection in the vertebrate host can modify the response to benznidazole. Exp Parasitol 118: 315–23. 17945216 10.1016/j.exppara.2007.08.016

[20] Gomes ML, Macedo AM, Vago AR, Pena SD, Galvão LM, Chiari E (1998) *Trypanosoma cruzi*: optimization of polymerase chain reaction for detection in human blood. Exp Parasitol 88: 28–33 9571142 10.1006/expr.1998.4191

[21] Avila H, Gonçalves AM, Nehme NS, Morel CM, Simpson L (1990) Schizodeme analysis of *Trypanosoma cruzi* stocks from South and Central America by analysis of PCR-amplified minicircle variable region sequences. Mol Biochem Parasitol 42: 175–87. 2270100 10.1016/0166-6851(90)90160-n

[22] Cummings KL, Tarleton RL (2003) Rapid quantitation of *Trypanosoma cruz*i in host tissue by real-time PCR. Mol Biochem Parasitol 129: 53–9. 12798506 10.1016/s0166-6851(03)00093-8

[23] Voller A, Bidwell DE, Bartlett A (1976) Enzyme immunoassays in diagnostic medicine: Theory and practice. Bull World Health Organ 53:55–65. 1085667 PMC2366417

[24] Nye FJ (1979) Treating toxoplasmosis. J Antimicrob Chemother 5:244–6 479059 10.1093/jac/5.3.244

[25] WHO. World Health Organization. (2001) Antimalarial drug combination therapy. Report of a WHO Technical Consultation.

[26] Lienhardt C, Raviglione M, Spigelman M, Hafner R, Jaramillo E, Hoelscher M, et al (2012) New drugs for the treatment of tuberculosis: needs, challenges, promise, and prospects for the future. Infect Dis 2:241–9. 10.1093/infdis/jis034 22448022

[27] Dybul ML, Fauci AS, Bartlett JG, Kaplan JE, Pau AK (2002) Panel on Clinical Practices for the Treatment of HIV Guidelines for using antiretroviral agents among HIV-infected adults and adolescents. Recommendations of the Panel on Clinical Practices for Treatment of HIV. MMWR Recomm Rep 51:1–55. 12027060

[28] Tarleton RL (2001) Parasite persistence in the aetiology of Chagas disease. Int J Parasitol 31:550–4 11334941 10.1016/s0020-7519(01)00158-8

[29] Bustamante JM, Rivarola HW, Fernandez AR, Enders JE, Fretes R, Palma JA, et al (2002) Trypanosoma cruzi reinfections in mice determine the severity of cardiac damage. Int J Parasitol 15: 889–896. 10.1016/s0020-7519(02)00023-1 12062560

[30] Caldas S, Caldas IS, Diniz LF, Lima WG, Oliveira R de P, Cecílio AB, et al (2012) Real-time PCR strategy for parasite quantification in blood and tissue samples of experimental *Trypanosoma cruzi* infection. Acta Tropica 123:170–177 10.1016/j.actatropica.2012.05.002 22609548

[31] Benvenuti LA, Roggério A, Freitas HF, Mansur AJ, Fiorelli A, Higuchi ML (2008) Chronic American trypanosomiasis: parasite persistence in endomyocardial biopsies is associated with high-grade myocarditis. Ann Trop Med Parasitol 102:481–7 10.1179/136485908X311740 18782487

[32] Lo Presti MS, Rivarola HW, Bustamante JM, Fernández AR, Enders JE, Fretes R, et al (2004) Thioridazine treatment prevents cardiopathy in *Trypanosoma cruzi* infected mice.Int J Antimicrob Agents 23:634–6 15194137 10.1016/j.ijantimicag.2003.10.006

[33] Garcia S, Ramos CO, Senra JF, Vilas-Boas F, Rodrigues MM, Campos de Carvalho AC, et al (2005) Treatment with benznidazole during the chronic phase of experimental Chagas' disease decreases cardiac alterations. Antimicrob Agents Chemother 49:1521–8. 15793134 10.1128/AAC.49.4.1521-1528.2005 PMC1068607

[34] Caldas IS, Talvani A, Caldas S, Carneiro CM, de Lana M, da Matta Guedes, PM, et al (2008) Benznidazole therapy during acute phase of Chagas disease reduces parasite load but does not prevent chronic cardiac lesions. Parasitol Res 103:413–21. 10.1007/s00436-008-0992-6 18454349

